# Societal vulnerability in the context of population aging—Perceptions of healthcare students' in Saudi Arabia

**DOI:** 10.3389/fpubh.2022.955754

**Published:** 2022-09-27

**Authors:** Osama A. Samarkandi, Mohammed Aljuaid, Mohammed Abdulrahman Alkohaiz, Ahmed M. Al-Wathinani, Abdullah Mohammed Alobaid, Abdullah A. Alghamdi, Mohammed A. Alhallaf, Nawaf A. Albaqami

**Affiliations:** ^1^Nursing Informatics, Department of Basic Sciences, Vice Dean for Academic Affairs, Dean of Prince Sultan Bin Abdulaziz College for Emergency Medical Services, King Saud University, Riyadh, Saudi Arabia; ^2^Department of Health Administration, College of Business Administration, King Saud University, Riyadh, Saudi Arabia; ^3^Social Studies Department, College of Arts, King Saud University, Riyadh, Saudi Arabia; ^4^Department of Emergency Medical Services (EMS), Prince Sultan College for Emergency Medical Services, King Saud University, Riyadh, Saudi Arabia; ^5^Department of Trauma and Accident, Prince Sultan Bin Abdulaziz College for Emergency Medical Services, King Saud University, Riyadh, Saudi Arabia; ^6^Department of Emergency Medical Services (EMS), Prince Sultan Bin Abdulaziz College for Emergency Medical Services, King Saud University, Riyadh, Saudi Arabia

**Keywords:** disaster, aging, healthcare students, societal vulnerability, Saudi Arabia

## Abstract

**Background and objective:**

Healthcare professionals have an important role in increasing awareness and protecting populations from natural disasters. This study aimed to assess the perception of healthcare students toward societal vulnerability in the context of population aging.

**Methods:**

This is a cross-sectional questionnaire-based study conducted among students from two different health colleges over 4 months from February to May 2021. Descriptive analysis was used to assess the perception, and inferential testing was used to assess the various association of knowledge toward societal vulnerability using SPSS.

**Results:**

The majority of respondents were male (69.2%), between 20 and 24 years of age (91.2%), and studying for a nursing degree (76.6%). Only 4.7% had previously completed a previous degree. The mean score of perceptions on the Aging and Disaster Vulnerability Scale among nursing students was 42.5 ± 10.3 (0–65) while for paramedicine 48.1 ± 9.7 (0–65). Similarly, the mean score among male students was 44.1 ±10.5. The mean PADVS total score for the cohort was 43.8 (SD = 10.5). The mean PADVS total score for nursing students was significantly lower than paramedic students (42.5 vs. 48.1; *p* < 0.001). There was no correlation between PADVS total score and gender, age, area of residence, or previous degree.

**Conclusion:**

Our results indicate that Saudi healthcare students perceive older adults are somewhat vulnerable to disasters with significant differences between nursing and paramedic students. Furthermore, we suggest informing emergency services disaster response planning processes about educational intervention to overcome disasters in Saudi Arabia and other countries.

## Introduction

Demographic and epidemiological transitions are defining features of geopolitics in the twenty-first century (1). It is estimated that by 2050, the global population of individuals aged 65 or older will reach 1.5 billion (1). The “oldest old” group, those aged 85 or over, is anticipated to increase by over 350% between 2010 and 2050, compared with an increase of just over 20 % among those aged under 65 (2). The projected growth in those 85 and older has significant implications for healthcare providers as advancing age is associated with chronic multi-morbidities and a range of geriatric syndromes, including frailty, functional decline, and cognitive impairment (2–4). An aging population will challenge paramedic service providers due to increased demand for attendance in community settings, more complex emergency department presentations, and transfers between residential care facilities and hospitals (5, 6). Increasing demand for paramedic services by persons aged over 85 has been identified as a significant workforce issue for the healthcare sector (6).

The Kingdom of Saudi Arabia (KSA) is a major economic power in the global economy experiencing accelerated economic and social change and an aging population (7, 8). Older people are recognized as being more vulnerable during and after disasters and suffer greater morbidity and mortality during major disasters. Evidence of the link between increasing extreme weather events and climate change suggests such events are likely to become more frequent, longer-lasting, and more intense in coming years (9, 10). Vicedo-Cabrera et al. found that during the period from 1991 to 2018, 37% of heat-related deaths could be attributed to anthropogenic climate change, and increased mortality was observed on all continents (11). Extreme weather events are predicted to have a greater impact on older community members (12).

Previous study in the USA reported that people aged over 60 comprised 70% of deaths resulting from Hurricane Katrina and almost 50% of deaths were reported among 75 years older. During the emergency and in the aftermath of that disaster, many older people were reportedly abandoned by their carer staff (13). Elsewhere, the research identified that in the wake of a catastrophic natural disaster in Japan many older people's Alzheimer's disease symptoms were exacerbated (14) and New Zealand researchers reported prevalent psychological problems among older adults in the aftermath of major earthquakes in that country (15).

The KSA is placed in the 84th highest position on the 2018 Global Climate Risk Index due to extreme heatwaves, repeated coastal flooding, shortage of freshwater sources, and vegetation cover of < 2% of the total land area (16). The country has experienced several natural and human-induced disasters in recent decades (17–20). Extreme weather events including heatwaves, floods, and dust storms caused considerable mortality, and economic cost, between 1980 and 2010 (17–20). In more recent times Middle East Respiratory Syndrome (MERS) and COVID-19 have further tested the capacity of health services across the country (19–21).

Emergency service providers are typically integral to a community's disaster response (22, 23). During disasters ambulance, call-outs, hospital transfers, and community mortality typically increase and have a significant impact on emergency service providers, including paramedics (24, 25). Paramedics serve multiple roles during disaster responses including triage, coordination of resources, and transport and evacuation of survivors (26–28). Many disaster victims are older people living in diverse settings and paramedic services typically address the immediate healthcare needs of this demographic (14). In the aftermath of a disaster, paramedics will often be required to manage frail older people with a range of age-related syndromes including functional and cognitive decline (29). Additionally, the ongoing care needs of this population do not subside in the aftermath of disasters and can be exacerbated during such times (30).

When assessing resilience and vulnerability to disasters older community members should be considered a distinct population concerning response and recovery planning. Multiple factors contribute to older adults being more susceptible to injury, illness, or death resulting from disasters (31, 32). Housing inhabited by older people is often constructed from older materials, and residing in such buildings is associated with a higher risk of death during extreme weather events (31, 33). Older people who are reliant on community and allied care services such as domestic support or food provision before a disaster have also been shown to be at higher risk of death (31, 34). People with reduced mobility have limited capacity for evacuation, and the presence of comorbidities also negatively influences health outcomes among older adults during disasters (31, 35). Further, the compromised heat adaptation and thermoregulatory capacity of older adults place them at additional risk in heatwaves (36). Therefore, this study aimed to assess the perception of healthcare students toward societal vulnerability in the context of population aging.

## Methods

### Design and setting

A cross-sectional study was conducted through self-administered questionnaires between February and May 2021. This study involves undergraduate paramedic and nursing students at King Saud University who were taught about disasters, undergo clinical practice experience, or had mass gathering experience in Hajj or Umrah season were eligible to participate. Participation was voluntary, and consent for the use of aggregated data in reporting was inferred by submission of the online survey. Ethics approval for this research was obtained from an institutional Human Research Ethics Committee (KSU-IRB 017E).

#### Survey tool development

The Perceptions of Aging and Disaster Vulnerability Scale (PADVS) is a tool for evaluating perceptions of vulnerability to disasters in the context of population aging (37). Following an extensive literature review, Annear et al. drafted 13 statements, all of which have been validated in Australia, Japan, and New Zealand settings (37). The Cronbach's α internal consistency reliability was 0.87 for the five subscales of the PADVS (37). The instrument was initially developed to enhance knowledge about the growing population aging and natural disasters in Japan. However, there is a dire need to better understand uses of the instrument in the Saudi Arabian context. Thus, given the potential application of this scale to healthcare students in Saudi Arabia, the instrument was adapted through rigorous forward-backward translation, face validity (38).

The data collection was carried out through google forms using online questionnaires, for the purpose of data collection, a researcher was appointed. The data collection was carried out using simple random sampling technique. The PADVS comprises 13 items, which are each scored from 0 to 5, based on respondents' perceptions of age-related societal vulnerability. An exemplar statement from the measure (item 1) reads as follows: “Societal vulnerability to natural disasters is increased by the growing number of older adults in the community.” Respondents scored each of the 13 statements from “0” (no increase in vulnerability) to “5” (very high increase in vulnerability). Within the 13-item measure are four validated subscales, which reflect different issues associated with age-related and societal vulnerability to disasters: (1) Isolation and access (4 items scored out of 20), (2) declining function (3 items scored out of 15), (3) community inclusiveness (3 items scored out of 15), and (4) health system and health worker readiness (3 items scored out of 15). Higher scores on the PADVS and each subscale indicate more significant concerns among respondents regarding societal vulnerability.

#### Data analysis

All data analyses were conducted using IBM SPSS for Mac (version 27). Continuous data were screened for outliers, and participants with standardized scores >3 or < -3 were excluded from further analysis. Descriptive statistics were reported for demographic characteristics, PADVS total score, and subscales scores. Correlations between PADVS total score and age, gender, prior education, and current degree were examined using the Pearson correlation or analysis of variance (ANOVA). Statistical significance was set at *p* < 0.05.

## Results

### Research participants

Of the 300 survey respondents, 295 were included in the analysis (Table 1). The majority of respondents were male (69.2%), between 20 and 24 years of age (91.2%), and studying for a nursing degree (76.6%). Only 4.7% had previously completed a previous degree. The mean score of perceptions on the Aging and Disaster Vulnerability Scale among nursing students was 42.5 ± 10.3, while for paramedicine 48.1± 9.7. Similarly, the mean score among male students was 44.1 ±10.5.

**Table 1:** Demographic characteristics of participants according to perceptions of aging and disaster vulnerability score.

**Variable**	**Population, % (*n*)**	**PADVS, total score, mean (SD)**	***p*-value*^a^***
**Gender**
Female	30.8% (91)	43.1 (10.5)	0.493
Male	69.2% (204)	44.1 (10.5)	
**Age**
Under 20	1.7% (5)	38.4 (11.8)	
20–24	91.2% (269)	44 (10.3)	
25–29	6.4% (19)	43.1 (12.4)	0.675
30 and over	0.7% (2)	42.5 (16.3)	
**Current degree**
Nursing	76.6% (226)	42.5 (10.3)	< 0.001
Paramedicine	23.4% (69)	48.1 (9.7)	
**Area of residence**
City	78.3% (231)	44.1 (10.6)	0.395
Rural	21.7% (64)	42.8 (9.9)	
**Previous degree**
No	95.3% (281)	44 (10.4)	0.196
Yes	4.7% (14)	40.3 (10.8)	
**Responses based on**
Wider Saudi Arabian society	61.7% (182)	44.8 (10.7)	0.045
Local community	38.3% (113)	42.3 (10.5)	

a p-value for differences in mean PADVS Total Score calculated using ANOVA. A p-value lower than 0.05 was considered statistically significant.

Table 2 detailed the student's frequency of perceptions of disaster vulnerability scale. The mean PADVS total score for the cohort was 43.8 (SD=10.5). The mean PADVS total score for nursing students was significantly lower than paramedic students (42.5 vs. 48.1; p < 0.001). There was no correlation between PADVS total score and gender, age, area of residence, or previous degree. Participants basing their responses on the wider Saudi Arabian society had a significantly higher PADVS total score than those considering their local community (44.8 vs. 42.3; *p* = 0.045). Respondents showed similar levels of concern across all four PADVS subscales (Table 3).

**Table 2:** Student's frequency of perceptions of disaster vulnerability scale.

**Questionnaire**	**0, *n* (%)**	**1, *n* (%)**	**2, *n* (%)**	**3, *n* (%)**	**4, *n* (%)**	**5, *n* (%)**
Q1. Societal vulnerability to natural disasters is increased by the growing	20 (6.6%)	22 (7.2%)	42 (13.8%)	88 (28.9%)	66 (21.7%)	66 (21.7%)
Q2. Societal vulnerability to natural disasters is increased by low incomes	06 (2.0%)	20 (6.6%)	43 (14.1%)	74 (24.3%)	76 (25.0%)	85 (28.0%)
Q3. Societal vulnerability to natural disasters is increased by limited participation of older adults in political decision making	22 (7.2%)	24 (7.9%)	54 (17.8%)	90 (29.6%)	57 (18.8%)	57 (18.8%)
Q4. Societal vulnerability to natural disasters is increased by living alone in later life	21 (6.9%)	19 (6.3%)	36 (11.8%)	77 (25.3%)	78 (25.7%)	73 (24.0%)
Q5. Societal vulnerability to natural disasters is increased by limited resilience of the built environment that serves older adults	13 (4.3%)	21 (4.9%)	41 (13.5%)	87 (28.6%)	73 (24.0%)	75 (24.7%)
Q6. Societal vulnerability to natural disasters is increased by limited access to social networks among older adults	15 (4.9%)	16 (5.3%)	56 (18.4%)	83 (27.3%)	60 (19.7%)	74 (24.3%)
Q7. Societal vulnerability to natural disasters is increased by limited access to information about disaster preparedness among older adults	9 (3.0%)	11 (3.6%)	51 (16.8%)	80 (26.3%)	71 (23.4%)	82 (27.0%)
Q8. Societal vulnerability to natural disasters is increased by declines in physical functions in later life	10 (3.3%)	24 (7.9%)	48 (15.8%)	84 (27.6%)	59 (19.4%)	79 (26.0%)
Q9. Societal vulnerability to natural disasters is increased by declines in sensory functions in later life	10 (3.3%)	16 (5.3%)	35 (11.5%)	83 (27.3%)	73 (24.0%)	87 (28.6%)
Q10. Societal vulnerability to natural disasters is increased by declines in cognitive functions in later life	9 (3.0%)	21 (6.9%)	38 (12.5%)	79 (26.0%)	81 (26.6%)	76 (25.0%)
Q11. Societal vulnerability to natural disasters is increased by low levels of health worker knowledge about the health problems of older adults	9 (3.0%)	16 (5.3%)	49 (16.1%)	96 (31.6%)	67 (22.0%)	67 (22.0%)
Q12. Societal vulnerability to natural disasters is increased by a lack of health workers Interprofessional collaboration in the care of older adults	9 (3.0%)	15 (4.9%)	58 (19.1%)	87 (28.6%)	68 (22.4%)	67 (22.0%)
Q13. Societal vulnerability to natural disasters is increased by insufficient health sector preparedness to care for older adults	12 (3.9%)	12 (3.9%)	53 (17.4%)	97 (31.9%)	59 (19.4%)	71 (23.4%)

**Table 3:** Absolute and comparative scores on 4 PADVS subscales addressing issues associated with age-related and societal vulnerability to disasters.

**PADVS Subscales**	**Mean**	**SD**	**Min**	**Max**	Normalized mean^*^
Isolation and access (scored out of 20)	13.6	3.8	0	20	0.68
Declining function (scored out of 15)	10.4	3.1	0	15	0.69
Community inclusiveness (scored out of 15)	9.8	2.9	0	15	0.65
Health system and worker readiness (scored out of 15)	10	3	0	15	0.67

* A higher score indicates greater respondent concern for issues relating to disaster vulnerability.

## Discussion

To our knowledge, this is the first study in Saudi Arabia that has investigated the Societal vulnerability in the context of population aging given the perceptions of healthcare students' in Saudi Arabia. Although not much literature was identified nationally about the perceptions of Societal vulnerability concerning aging. However, most of the literature reported internationally on the context of vulnerability of aging (37, 39). This study would make a substantial contribution to protecting populations from natural disasters, especially concerning the effects of climate change in Saudi Arabia, and would serve as a reference for future studies that are desperately needed.

In this study, the mean PADVS total score among Saudi healthcare students was 43.8, which is slightly inconsistent with previous studies conducted in other countries (37, 39). For instance, an earlier study conducted among Australian healthcare students reported a total score of perceptions was 45.39 (range 0–65). The difference in the findings might be due to the differences in the study site, population, and weather of the country or country profile where the participants live. Furthermore, it is evidenced that islands occupied by oceans, the possibilities of frequent innocence of natural disasters (37, 39). This might be the reason for a higher understanding of disasters and their consequences in previous studies, where earthquakes, tsunamis, and typhoons are common, compared to an arid country like Saudi Arabia.

Similarly, in the current study, the mean overall score for the isolation and access, declining function, community inclusiveness, and health system and worker readiness was 13.6 ± 3.8, 10.4 ± 3.1, 9.8 ± 2.9, 10 ± 3. While the mean score for the domains of isolation and access, the declining function was reported poorly in comparison to a previous study by Lucas et al. in 2019, who reported 14.28 ± 3.70, 12.58 ± 2.48 (51). However, the community inclusiveness and the domain of health system worker readiness scores in this study are better than the previous study conducted by Lucas et al. in 2019 (39). This indicates that this topic should be given more attention in Saudi healthcare education programs, and other international levels, as community resilience to disaster is dependent on active engagement and awareness (39, 40). Furthermore, our research reveals a lack of awareness regarding disaster vulnerability in the context of an aging population in Saudi Arabia.

In this study, the perception score was high among paramedical students [48.1 (9.7)] compared to nursing students [42.5 (10.3)]. However, evidence was shown that paramedics are well-trained to address the health problems faced by older adults during and after a natural disaster. In the typically chaotic aftermath of a big disaster, health and social care workers must be able to function successfully in times of social and environmental disturbance (39). Similarly, the perception scores were significantly different between students belonging to Wider Saudi Arabian society and the local community. In Saudi Arabia, where a significant number of older people live in holy cities (41) and the risk of tropical diseases and fire, is exacerbated as a result of annual pilgrimages visits to holy cities and also due to extreme weather events, the vulnerability of older adults is also likely to increase (42). Furthermore, in Saudi Arabia, increased temperatures in the Summer may increase the risks of heat-related mortalities.

### Limitations

The current study has some limitations, first, the research involved students from one campus of a large public university in the capital of Saudi Arabia. Consequently, results may not be more broadly generalizable at this stage and or not-representative of others, as well as being not generalizable globally. However, the distribution in the data and a diversity of perspectives among the sample suggest that the findings may be replicated with larger random samples. Second, the results were based on a self-completed questionnaire, which may have increased the possibility of biases such as social desirability bias or recall bias.

### Recommendation

The findings of this study recommend that disaster vulnerability be established a central part of geriatric education for aspiring healthcare students particularly paramedics and nurses. In order to inform future initiatives aimed at increasing awareness of societal vulnerability to disaster in the context of an aging population, we urge that outcomes of any such educational programs be extensively examined.

## Conclusion

Our results indicate that Saudi healthcare students perceive older adults are somewhat more vulnerable to disasters with significant differences between nursing and paramedic students. Our findings suggest that current healthcare professional education does not adequately prepare students to overcome the natural disasters, which are expected to become more frequent and extreme as the Geological era progresses. Furthermore, it is advisable to inform emergency services about disaster response planning processes and educational intervention.

## Data availability statement

The raw data supporting the conclusions of this article will be made available by the authors, without undue reservation.

## Ethics statement

Ethics approval for this research was obtained from an institutional Human Research Ethics Committee, King Saud University No. KSU-IRB 017E. The patients/participants provided their written informed consent to participate in this study.

## Author contributions

Conceptualization, validation, formal analysis, investigation, supervision, and project administration: OS, MAb, MAlj, and AA-W. Methodology, data curation, and writing—original draft preparation: AAlo, NA, MAlh, and AAlg. Software: OS and NA. All authors reviewed the manuscript, contributed to the article, and approved the submitted version.

## Funding

MA received a fund from the Researcher Supporting Project number (RSP2022R481), King Saud University, Riyadh, Saudi Arabia, to support the publication of this article. The funding agency had no role in designing the study, conducting the analysis, interpreting the data or writing the manuscript.

## Conflict of interest

The authors declare that the research was conducted in the absence of any commercial or financial relationships that could be construed as a potential conflict of interest.

## Publisher's note

All claims expressed in this article are solely those of the authors and do not necessarily represent those of their affiliated organizations, or those of the publisher, the editors and the reviewers. Any product that may be evaluated in this article, or claim that may be made by its manufacturer, is not guaranteed or endorsed by the publisher.
